# Provision of Clinical Preventive Services by Community Pharmacists

**DOI:** 10.5888/pcd13.160232

**Published:** 2016-10-27

**Authors:** Sarah E. Kelling, Angela Rondon-Begazo, Natalie A. DiPietro Mager, Bethany L. Murphy, David R. Bright

**Affiliations:** Author Affiliations: Angela Rondon-Begazo, University of Michigan College of Pharmacy, Ann Arbor, Michigan; Natalie A. DiPietro Mager, Ohio Northern University Raabe College of Pharmacy, Ada, Ohio; Bethany L. Murphy, Union University School of Pharmacy, Jackson, Tennessee; David R. Bright, Ferris State University College of Pharmacy, Big Rapids, Michigan.

## Abstract

Community pharmacists are highly accessible health care professionals, providing opportunities for partnerships with other health care and public health professionals to expand the population’s access to clinical preventive services. To document examples of the community pharmacist’s role in providing clinical preventive services to the general population, we conducted PubMed searches using the key word “community pharmacy” and key words from the US Preventive Services Task Force recommendations rated A or B. We present 4 descriptive summaries of clinical preventive services that can be offered by community pharmacists. Community pharmacists can provide clinical preventive services such as providing education, conducting screenings, and making referrals to improve population health.

## Introduction

Preventive health care aims to prevent disease from occurring (primary prevention), reduce progression of disease by identifying it before it becomes symptomatic (secondary prevention), and decrease the impact of disease if it does occur (tertiary prevention). Data about the clinical utility of primary and secondary prevention services are multiple and often conflicting, with various recommendations for use by health care providers. The US Preventive Services Task Force (USPSTF) is one expert panel that provides guidance to clinicians on the effectiveness of clinical preventive services based on systematic reviews of the literature. Preventive services labeled as A or B indicate that the USPSTF recommends the service and suggests that clinicians offer or provide these services to patients (1).

Historically in the United States, overall use of preventive health services has been low, especially among racial and ethnic minorities, people who are uninsured, and people who have low incomes (2,3). Increased attention has been given to preventive health care in recent years because of implementation of the Affordable Care Act, which requires that most health plans cover A and B recommendations with no cost-sharing to the patient (4). Approximately 100,000 lives could be saved in the United States each year with widespread adoption of just 5 recommendations: daily aspirin use for adults with cardiovascular disease risk, advising smokers to quit and offering assistance in quitting, annual influenza vaccine for patients aged 50 years or older, colorectal cancer screening for adults aged 50 years or older, and breast cancer screening for women aged 40 years or older (2).

Pharmacists are recognized as providers of preventive health care services (5,6). Community pharmacists may be particularly well positioned to increase appropriate use of services, either through direct delivery of services or by providing education and referrals. Because 90% of Americans live within 5 miles of a community pharmacy, there is opportunity for other health care and public health professionals to partner with community pharmacists to expand the population’s access to clinical preventive services (7). The purpose of our review was to document the community pharmacist’s role in providing clinical preventive services to men and nonpregnant women aged 18 years or older in the United States. Through providing select examples of community pharmacists implementing USPSTF A and B recommendations, we hope that more public health and health care professionals will realize the potential of pharmacists to expand access to clinical preventive services and that additional ideas for collaboration will be stimulated.

## Methods

All 95 USPSTF recommendations were reviewed in March 2015, and those that were rated A or B and that focused on services for nonpregnant adults were considered for inclusion in the study. We searched PubMed using the key word “community pharmacy” and key words from the US Preventive Services Task Force recommendations rated A or B to identify studies demonstrating implementation of clinical preventive services by community pharmacists in the United States. Based on the expertise of the authors and a review of published literature, recommendations were categorized as either opportunities for community pharmacists to conduct the service within pharmacies or to provide education and outside referral.

From the published literature that was identified, we selected articles that represented various services across different types of community pharmacies. The intention of the article selection process was not to create an exhaustive listing or review of service models but to provide a diverse representation of services in the literature to spur discussion and thought about how to further incorporate pharmacists into preventive care. Because the USPSTF does not make its own recommendations about vaccinations and a substantial literature already documenting pharmacist impact exists in this area, articles focused on pharmacist provision of vaccinations were excluded from review.

Of the 95 USPSTF recommendations reviewed, 32 were rated as A or B recommendations and focused on nonpregnant adults (Figure). A total of 18 recommendations were categorized as opportunities for community pharmacists to conduct the service (Table 1) and 14 recommendations were categorized as opportunities for community pharmacists to provide education or referrals (Table 2). Pharmacist implementation of interventions that were focused on folic acid education, tobacco use cessation, osteoporosis screening, and HIV screening were selected for discussion here.

**Figure:**
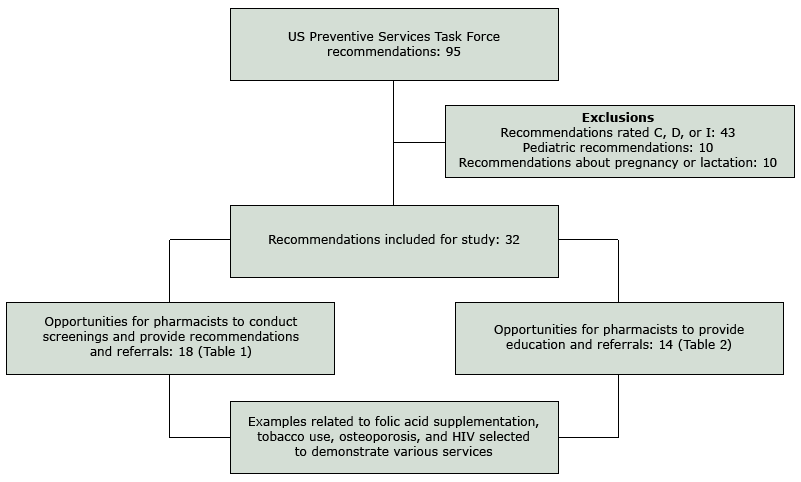
Determination of US Preventive Services Task Force recommendations to review with regard to provision of clinical preventive services by community pharmacists. The US Preventive Services Task Force states that recommendations with a C rating be offered or provided to selected patients based on individual circumstances, D-rated recommendations should be discouraged, and I-rated recommendations have insufficient evidence to assess the balance of benefits and harms for the service.

**Table 1:** Opportunities in the Recommendations of the US Preventive Services Task Force for Community Pharmacists to Conduct Screenings and Provide Recommendations and Referrals for Health Care, 2015

Recommendation (Activity)	Population
**Chronic diseases**	
Blood pressure in adults (screening)	Adults aged 18 years or older
Cholesterol abnormalities^a^ (screening)	Men aged 20–35 years at increased risk for coronary heart disease, all men aged 35 years or older, and women aged 20 years or older at increased risk for coronary heart disease
Diabetes mellitus (screening)	Asymptomatic adults with sustained blood pressure (treated or untreated) greater than 135/80 mm Hg
**Conditions affecting older adults**	
Aspirin for the prevention of cardiovascular disease (preventive medication)	Men aged 45–79 years when the potential benefit due to the reduction in myocardial infarctions outweighs the potential harm of gastrointestinal hemorrhage and women aged 55–79 years when the potential benefit due to the reduction in ischemic strokes outweighs the potential harm of gastrointestinal hemorrhage
Fall prevention in older adults (counseling, preventive medication, and other interventions)	Community-dwelling adults aged 65 years or older who are at increased risk for falls
Osteoporosis (screening)	Women aged 65 years or older and younger women whose fracture risk is equal to or greater than that of a 65-year-old white woman who has no additional risk factors
**Healthy behaviors**	
Alcohol misuse screening and behavioral counseling (screening and counseling)	Adults aged 18 years or older
Depression in adults (screening)	Adults aged 18 years or older
Folic acid supplementation to prevent neural tube defects (preventive medication)	All women planning or capable of pregnancy
Healthy diet and physical activity counseling to prevent cardiovascular disease (counseling)	Adults who are overweight or obese and have additional cardiovascular disease risk factors
Intimate partner violence (screening)	Women of childbearing age
Obesity in adults (screening and counseling)	Adults aged 18 years or older
Tobacco use in adults (counseling and interventions)	Adults aged 18 years or older
**Infectious diseases**	
Hepatitis C virus infection (screening)	People at high risk for infection and adults born during 1945–1965
HIV infection (screening)	People aged 15–65 years, younger adolescents, and older adults at increased risk

a Four separate age-based and sex-based recommendations have been summarized into this category.

**Table 2:** Opportunities in the Recommendations of the US Preventive Services Task Force for Community Pharmacists to Provide Education and Referrals for Health Care, 2015

Recommendation (Activity)	Population
**Cancer**	
*BRCA*-related cancer in women (screening)	Women with a family history of breast cancer and who are at low risk for adverse medical effects
Breast cancer (preventive medication)	Women at increased risk for breast cancer and low risk for adverse medical effects
Breast cancer (screening)	Women aged 40 years or older
Cervical cancer (screening)	Women aged 21–65 years
Colorectal cancer (screening)	Adults aged 50–75 years
Lung cancer (screening)	Adults aged 55–80 years who have a 30 pack-year^a^ smoking history and currently smoke or have quit within the past 15 years
Skin cancer (counseling)	People aged 10–24 years who have fair skin
**Conditions affecting older adults**	
Abdominal aortic aneurysm (screening)	Men aged 65–75 years who have ever smoked
Fall prevention in older adults (exercise or physical therapy)	Community-dwelling adults aged 65 years or older at increased risk for falls
**Infectious diseases**	
Chlamydia (screening)	Sexually active women aged 24 years or younger and older women at increased risk for infection
Gonorrhea (screening)	Sexually active women aged 24 years or younger and older women at increased risk for infection
Hepatitis B (screening)	People at increased risk
Sexually transmitted infections (counseling)	All sexually active adolescents and adults at increased risk
Syphilis (screening)	People at increased risk

a The number of packs of cigarettes smoked per day multiplied by the number of years the person has smoked (eg, 1/2 pack per day × 10 years = 5 pack-year history; 2 packs per day × 10 years = 20 pack-year history).

## Primary Prevention

### Preventive medication: folic acid supplementation

Community pharmacists are well-situated to counsel women of childbearing potential about folic acid for the prevention of neural tube defects (NTDs). Although NTDs affect approximately 3,000 pregnancies each year in the United States, 50% to 70% of NTDs are preventable by consuming adequate amounts of folic acid daily before conception and during the first trimester of pregnancy (8,9). Because many women do not consume the necessary amounts of folic acid through diet alone and there is a high rate of unplanned pregnancies in the United States, supplementation in the form of a folic acid tablet or multivitamin containing folic acid is recommended for all women of childbearing potential, regardless of pregnancy intention (1). For most women of childbearing potential, 400 μg (0.4 mg) is the recommended daily dose. Women with certain risk factors should be advised to take higher doses, including women with diabetes (usually 4–5 mg/d), using anti-epileptic drugs (usually 4 mg/d), or who have experienced a previous NTD-affected pregnancy (usually 4 mg/d) (10).

Many American women are still not aware of the need for daily folic acid intake to prevent NTDs (11). In addition, data indicate that health care professionals are not consistently informing women about this need (11). However, nearly 90% of women responding to a March of Dimes survey indicated that they would be likely to take a daily folic acid supplement if advised to do so by a health care provider (11).

There are many ways that community pharmacists can fill these unmet needs. Education interventions, such as a live educational program led by pharmacists and student pharmacists result in statistically significant improvements in knowledge and increased use of folic acid by participants (12–14). In addition, recommendations for folic acid can be addressed through medication therapy management (10). Finally, pharmacists can use promotional materials such as posters and brochures in their pharmacies to raise awareness about folic acid and initiate conversations with patients. National observances each January for National Birth Defects Prevention Month and Folic Acid Awareness Week and in March for World Birth Defects Day provide further opportunities to promote folic acid use among patients in the community pharmacy.

## Secondary Prevention

### Healthy behaviors: tobacco use

The USPSTF recommends that all adults be asked about tobacco use and be provided cessation interventions if they use tobacco products (1). The 2014 National Health Interview Survey (NHIS) reported a significant reduction in tobacco smoking from 20.9% (45.1 million people) in 2005 to 16.8% (40.0 million people) in 2014 (15). Proven population-based interventions (tobacco price increases, smoke-free laws, media campaigns, increased access to smoking cessation programs) are producing positive outcomes; however, the *Healthy People 2020* objective is to reduce the prevalence of tobacco smoking to less than 12% (15). Among the surveyed individuals in the NHIS, people who live below the federal poverty level, have a General Educational Development (GED) certificate, are racial/ethnic minorities, are nonstraight adults, are uninsured, and have only Medicaid had a high smoking prevalence. Community pharmacists are uniquely positioned to assist in attaining the *Healthy People 2020* objective because of their training and their accessibility to vulnerable populations. Furthermore, engagement of multiple health professionals increases readiness to quit smoking, and studies have demonstrated success in the community pharmacy setting (16).

Common barriers to providing tobacco use cessation services in community pharmacies include time limitations, lack of training, and pharmacist self-efficacy. The Ask, Advise, Refer (AAR) model (17) addresses the time barrier. It requires less than 5 minutes and allows for prompt and short interventions with the assistance of pharmacy technicians. Patwardhan and Chewning randomly assigned 16 high-volume chain pharmacies to either the intervention (use of AAR, tobacco use cessation posters, and pharmacy technician involvement in the Ask and Fax-to-Quit enrollment steps) or control (provide quitline cards only) groups (17). A significantly higher number of patients in the intervention groups were asked about tobacco use, advised to quit, and enrolled in the quitline. Frameworks such as the AAR, AAC (Ask, Advice, Connect), and the 5 A’s (Ask, Advise, Assess, Assist, Arrange) provide feasible means for community pharmacists to assist patients in tobacco use cessation (17).

### Chronic disease screening: osteoporosis

The USPSTF recommends that women aged 65 years or older, as well as younger women at a high risk of fractures, be screened for osteoporosis (1). There are many risk factors for osteoporosis, and the FRAX tool (https://www.shef.ac.uk/FRAX/) can be used to assess an individual’s 10-year risk of osteoporotic fracture based on age, sex, presence of rheumatoid arthritis or secondary causes of osteoporosis, fracture history, family history of fracture, bone mineral density, smoking status, body mass index, alcohol use, and glucocorticoid use. Because osteoporosis is an asymptomatic disease that affects more than 10.2 million Americans (29) and can lead to fractures resulting in substantial illnesses and deaths, screening services are important for early identification of this bone abnormality to ensure provision of appropriate treatment and reduction in fracture risk.

Although dual-energy X-ray absorptiometry (DXA) remains the gold standard for diagnosing osteoporosis, quantitative ultrasonography (QUS) provides a less expensive, more readily accessible alternative for initial screening of individuals (1). Although it cannot be used to diagnose osteoporosis, QUS of the calcaneus allows for prediction of fracture risk of the femoral neck, hip, and spine, and is frequently used to identify people who should receive follow-up screening with DXA for potential diagnosis (1).

Because of its portability, comparatively low cost, and ease of use, QUS provides pharmacists in the community setting an opportunity to improve rates of screening for osteoporosis. Several models have been published that illustrate community pharmacists screening patients for osteoporosis (18–20). In some, patients self-identified the need for screening based on perceived risk; other involved referrals by physicians from various clinical settings (19–21).

### Infectious disease screening: HIV

The opportunity to screen for chronic infectious diseases, such as HIV, has been demonstrated in community pharmacies (22). Approximately 240,000 people with HIV in the United States are undiagnosed (23). Early diagnosis may lead to early treatment and reduced disease spread. Testing can be conducted at community pharmacies to increase access to care (22–24).

The Centers for Disease Control and Prevention completed a pilot in which confidential HIV testing was offered at 21 sites across the United States, including 18 community pharmacies (24). Community pharmacies can serve as a HIV testing sites with specific care linkages for referral for confirmatory testing and clinical support (23). When this service was implemented in 2 community pharmacies in Michigan, there was a similar testing rate (1.5%) (22). That study also found that pharmacists felt comfortable performing the testing.

The Food and Drug Administration approved the first oral HIV home test kit that can be purchased in pharmacies across the country (25). Pharmacists interviewed in one study suggested that consultation and counseling would be helpful when the over-the-counter HIV test is purchased (eg, discussion of test procedure, building a relationship so that future consultation could occur if desired) (25). Community pharmacists can also provide appropriate counseling related to disease education and prevention strategies (eg, safe sex strategies, injection and syringe habits).

## Counseling and Referral

Community pharmacists are well suited to provide education and referrals for community members in need of clinical preventive services that are not routinely offered at pharmacies or that pharmacists usually do not perform. These services could include screenings that the pharmacy chooses not to provide (eg, are not cost-effective, the pharmacy staff are not trained to provide) or screenings that are not feasible in the community pharmacy setting (eg, screenings requiring specialized equipment or expertise). Pharmacists can identify patients who are candidates for specific screenings, provide education to the patient, and if appropriate make a recommendation about where to receive the screening. Examples of USPSTF recommendations that community pharmacists could provide education and referrals for may include screenings for abdominal aortic aneurysms, *BRCA* genetic testing, colorectal cancer, and depression.

## Discussion

Community pharmacists are increasingly being recognized as potential partners in many public health activities. Pharmacists have demonstrated their utility in many areas, including chronic disease management (6,7). Similarly, community pharmacists can improve population health through the provision of clinical preventive services. To expand population coverage for clinical preventive services, public health and health care professionals should consider including community pharmacies because of their expertise and unique accessibility. Community pharmacies offer convenient hours and locations for millions of Americans and, as a result, can provide critical access to these services for vulnerable populations and those considered hard to reach (8). The development of clinical–community linkages, whereby public and private sectors collaborate, helps to increase patient access to various services by increasing coordination of services, identifying and closing gaps in services, and encouraging community members to become involved in improvement initiatives (26).

Community pharmacists who provide clinical preventive services often need to build collaborative partnerships with other health care professionals. Depending on the state’s laws, pharmacists may be able to provide clinical preventive services directly to patients without direct oversight of a physician or other health care professional. However, developing partnerships helps to ensure that patients who are identified as having high risk (eg, positive depression screening) have access to appropriate follow-up services (eg, cognitive behavioral therapy, pharmacotherapy). In some situations, it may be helpful for the community pharmacy to have a collaborative practice agreement, which would allow an expanded scope of practice. Although state laws vary, in general a collaborative practice agreement may authorize pharmacists to order laboratory testing, to start or change drug therapy, or to do other duties through formal collaboration with a prescriber. Furthermore, health care professionals may find value in referring their patients to pharmacy-based services, particularly those services which are time consuming (eg, healthy diet and physical activity counseling). Strategies that also may help to create partnerships include meeting face-to-face to develop rapport between the different health care professionals and working toward a shared electronic health record to effectively communicate about patient care (27,28). Pharmacists may be able to be reimbursed for these services directly or indirectly, given their practice site and scope of practice.

## Conclusion

Clinical preventive services offer the opportunity to improve the health of populations and decrease health care costs. Community pharmacists have demonstrated the ability to implement USPSTF recommendations, either through screening, education, and recommendations to patients (eg, folic acid supplementation, tobacco use cessation) or screening and referrals to primary care providers for follow up testing and care (eg, osteoporosis screening, HIV screening). We provide a summary of services that community pharmacists can offer as well as selected examples of interventions addressing USPSTF A and B recommendations. As part of interdisciplinary teams and clinical–community links, community pharmacists can improve population health through provision of clinical preventive services.
