# Addendum, Vol. 13, October 27 Release

**DOI:** 10.5888/pcd13.160232e

**Published:** 2016-12-01

Information about funding source was omitted from the Acknowledgments section of the article “Provision of Clinical Preventive Services by Community Pharmacists,” which was released on October 27, 2016. This research received no specific grant from any funding agency in the public, commercial, or nonprofit sectors. The statement was added to the article on November 16, 2016.

The corrected article appears online at http://www.cdc.gov/pcd/issues/2016/16_0232.htm. We regret any inconvenience this omission may have caused.

